# A post hoc analysis of Projected Retained Ability Scores (PRAS) for the longitudinal assessment of cognitive functioning in patients with neuronopathic mucopolysaccharidosis II receiving intrathecal idursulfase-IT

**DOI:** 10.1186/s13023-023-02957-2

**Published:** 2023-11-02

**Authors:** Karen S. Yee, Costel Chirila, Eric Davenport, Deirdre Mladsi, Christine Barnett, William G. Kronenberger

**Affiliations:** 1 Takeda Development Center Americas, Inc., Cambridge, MA USA; 2 RTI Health Solutions, Research Triangle Park, NC USA; 3 Indiana University School of Medicine, Indianapolis, IN USA; 4 Present Address: Alexion Pharmaceuticals, Inc., AstraZeneca Rare Disease, Boston, MA USA

**Keywords:** Mucopolysaccharidosis II, Hunter syndrome, Idursulfase, Neuronopathic, Cognitive development, Projected Retained Ability Score

## Abstract

**Background:**

Norm-based scores used to assess cognitive ability have clinical value when describing functioning of patients with neuronopathic disorders compared with unaffected, same-age peers. However, they have limitations when used to assess change in cognitive ability between two timepoints, especially in children with severe cognitive decline. Calculation of Projected Retained Ability Scores (PRAS) is a novel method developed to characterize absolute change in norm-based ability test scores. In this analysis, PRAS were calculated post hoc for children with mucopolysaccharidosis II (MPS II; Hunter syndrome) and early cognitive impairment in a 52-week phase 2/3 randomized controlled trial (RCT) and its extension study of intrathecal idursulfase (idursulfase-IT). Patients completing the first year of the extension after receiving idursulfase-IT in the RCT and extension (n = 32 of 34 enrolled) or the extension only (n = 15 of 15 enrolled) were categorized according to changes in Differential Ability Scales, Second Edition, General Conceptual Ability (DAS-II GCA) scores and PRAS at 1 and 2 years. Analyses were conducted in the overall population and a subpopulation aged < 6 years at baseline (idursulfase-IT in the RCT and extension [n = 27] and extension only [n = 12]).

**Results:**

PRAS methodology differentiated patients with decreases in DAS-II GCA scores into three separate categories reflecting below-average cognitive growth rates, plateauing cognitive development, and deteriorating cognitive functioning. After 1 year in the RCT, 72.4% of patients who initiated idursulfase-IT had above-average or average cognitive growth rates in DAS-II GCA scores compared with 53.3% of those who did not receive idursulfase-IT; 6.9% versus 20.0% experienced deteriorating cognitive functioning. Similar results were seen in children aged < 6 years: 76% (idursulfase-IT group) versus 50% (no idursulfase-IT) had above-average or average cognitive growth rates in DAS-II GCA scores; 4% versus 17% had deteriorating cognitive functioning. The difference in the distributions of cognitive categories at 1 year in children aged < 6 years was significant (*p* = 0.048). At 2 years, the proportions of patients in different cognitive categories were more similar between treatment groups.

**Conclusions:**

PRAS methodology may help to differentiate changes in cognitive development in MPS II, and therefore may represent a valuable addition to existing approaches for interpreting changes in cognitive scores over time.

*Trial Registration*: ClinicalTrials.gov NCT02055118 (registration date: 4 February 2014) and NCT02412787 (registration date: 9 April 2015).

**Supplementary Information:**

The online version contains supplementary material available at 10.1186/s13023-023-02957-2.

## Background

Mucopolysaccharidosis II (MPS II; Hunter syndrome; OMIM 309900) is a rare, X-linked lysosomal storage disease with an estimated worldwide prevalence of between 1 in 100,000 and 1 in 170,000 male live births [1–8]. MPS II is caused by deficient activity of the iduronate-2-sulfatase (I2S) enzyme, which leads to accumulation of glycosaminoglycans throughout the body, resulting in progressive, multisystemic clinical signs and symptoms [8]. Common signs and symptoms include hepatosplenomegaly, respiratory disease, cardiovascular involvement, hearing loss, and joint and muscle disorders [2, 8]. However, clinical presentation and disease course are highly variable [2, 7, 8]. Traditionally, MPS II has been classified by the presence or absence of central nervous system involvement and cognitive impairment or neurocognitive decline in addition to the somatic manifestations [8]. It is becoming clear, however, that MPS II presents on a spectrum and there is substantial variability in the disease trajectory with differences in timing of the slowing and decline of cognitive development [9, 10].

The current standard of care for MPS II is intravenous enzyme replacement therapy with recombinant I2S (idursulfase; Takeda Pharmaceuticals USA, Inc., Lexington, MA, USA), which has been shown to be effective in the treatment of somatic manifestations of the disease and in improving survival [9, 11–15]. However, intravenous idursulfase has not been shown to cross the blood–brain barrier at levels sufficient to abate cognitive decline [16–18]. To address this, recombinant idursulfase formulated for intrathecal administration (idursulfase-IT) via an intrathecal drug delivery device was developed [19]. A phase 2/3 trial (HGT-HIT-094; NCT02055118) and open-label extension (SHP609-302; NCT02412787) evaluated the safety and efficacy of idursulfase-IT for the treatment of neuronopathic MPS II [20, 21]. The primary efficacy endpoint of the phase 2/3 trial was the Differential Ability Scales, Second Edition (DAS-II) General Conceptual Ability (GCA) score, which measures cognitive function relative to a same-age normative sample [22]. Although the primary endpoint was not met, there was a trend towards a treatment benefit measured by cognitive assessment scores, particularly in patients younger than 6 years of age at baseline with missense I2S gene (*IDS*) variants [20]. In the extension study, during which all patients received idursulfase-IT, the benefit of early treatment with idursulfase-IT as measured by cognitive assessment scores in patients younger than 6 years of age was maintained for 36 months [21].

Global ability scores are used to assess broad cognitive ability on major intellectual tests such as the Wechsler scales, DAS, Bayley, and Kaufman ability scales [22–25]. They are expressed as norm-based scores, which provide information about a child’s cognitive ability in the context of a same-age representative normative sample drawn from the population. However, they have limitations when used to assess change in cognitive ability between two timepoints in individual young children with a condition such as neuronopathic MPS II, in which cognitive function is not expected to progress at the same rate as in healthy same-age peers [26]. A child whose cognitive ability improves from timepoint one to timepoint two at the same rate as same-age peers will show no change in norm-based cognitive ability score (‘average cognitive growth rate’). There is a particular challenge when norm-based scores decline between two timepoints [27]. In such cases, it is not possible to ascertain whether the child has shown an absolute improvement in cognitive functioning (albeit at a slower rate than that of same-age peers; ‘below-average cognitive growth rate’), no change in cognitive functioning (which would lead to a decline in norm-based scores relative to the age-typical cognitive improvement shown in the normative sample; ‘plateauing cognitive functioning’), or a decline in absolute cognitive functioning (and loss of milestones) relative to the child’s cognitive ability at the previous timepoint (‘deteriorating cognitive functioning’) [26]. Thus, norm-based scores such as DAS-II GCA scores cannot differentiate between absolute change in the form of growing (albeit slower than norm peers), plateauing, or deteriorating cognitive function in individual young children over time. This information is important for understanding disease progression and the potential impact of treatment in children with neurodegenerative conditions such as MPS II, because cognitive growth (even if it is below-average) indicates continued developmental progress, whereas deteriorating cognitive functioning suggests neurodegeneration.

To address this fundamental limitation of using norm-based scores in isolation, the Projected Retained Ability Score (PRAS) methodology has been developed to characterize the absolute change (i.e., change in absolute performance in the same child from baseline to endpoint) in norm-based scores over time. When applied to DAS-II GCA scores, PRAS allows differentiation between below-average cognitive growth rates versus plateauing and deteriorating (i.e., loss of milestones) cognitive functioning in children with MPS II [26]. The aim of this post hoc analysis was to assess further the value of PRAS as a tool to assess cognitive function changes in patients with neuronopathic MPS II by applying PRAS methodology to the DAS-II GCA scores of patients treated with idursulfase-IT in the phase 2/3 trial and extension study.

## Materials and methods

### Study design

The designs of the phase 2/3 trial and extension study have been described previously [20, 21]. In brief, the phase 2/3 trial was a controlled, randomized, two-arm, open-label, assessor-blinded, multicenter study, in which patients aged from ≥ 3 to < 18 years with MPS II and early cognitive impairment were randomly assigned in a 2:1 ratio to receive monthly idursulfase-IT 10 mg or no idursulfase-IT treatment for 1 year, in addition to weekly intravenous idursulfase 0.5 mg/kg. Patients who completed the 52-week assessment of the phase 2/3 trial were eligible to participate in an open-label, non-randomized extension study, in which all patients received monthly idursulfase-IT 10 mg in addition to intravenous idursulfase 0.5 mg/kg. In the extension study, patients assigned to idursulfase-IT in the phase 2/3 trial were defined as the early idursulfase-IT group. Patients assigned to no idursulfase-IT treatment in the phase 2/3 trial who initiated treatment with idursulfase-IT in the extension study were defined as the delayed idursulfase-IT group.

### Endpoints and assessments

In the phase 2/3 trial, the primary endpoint was the change from baseline in DAS-II GCA score at week 52 [20]. DAS-II GCA was assessed at baseline and weeks 16, 28, 40, and 52.

The primary objective of the extension study was to assess long-term safety; long-term clinical efficacy measures were also assessed [21]. The primary efficacy outcome for the extension was change from phase 2/3 baseline in DAS-II GCA score.

### Application of PRAS and differentiation of patients into cognitive development categories

For this post hoc analysis, PRAS methodology was applied to DAS-II GCA scores for all randomized patients with available data at baseline and at 1 year and 2 years (the overall population). In addition, this analysis was conducted for the subpopulation of patients aged younger than 6 years at phase 2/3 baseline, who were the focus of the efficacy analyses of the extension study [21].

For each patient, the PRAS GCA scores at year 1 and year 2 were derived by applying the baseline GCA subtest raw/ability score to the norms for the patient’s age at follow-up (year 1/year 2), to provide a projected score (Additional file 1: Fig. S1) reflecting the expected follow-up GCA score that would have been obtained if the patient had achieved exactly the same raw score as at baseline [26]. The PRAS GCA score (year 1/year 2) was then compared with the measured follow-up GCA score (year 1/year 2) to evaluate the absolute change. Based on the standard error of measurement and standard error of the difference of DAS GCA scores, a difference of > 10 points between endpoint GCA score and baseline GCA score (relative change) or between endpoint GCA score and PRAS (absolute change) was considered clinically and statistically meaningful [26].

For patients who transitioned from the Early Years battery of the DAS-II to the School Age battery during the study and were younger than 9 years of age when they completed the assessment, PRAS values were calculated by applying the extended age range norms for 7 years, 0 months, to 8 years, 11 months, to baseline scores for the Early Years battery subtests. Those PRAS scores were then compared with endpoint GCA scores from the School Age battery. For patients who transitioned from the Early Years battery of the DAS-II to the School Age battery during the study and were 9 years of age or older when they completed the assessment, the PRAS could not be calculated owing to lack of extended age norms after age 8 years, 11 months.

Patients were grouped into one of five cognitive categories based on baseline GCA score, endpoint GCA score, and PRAS (Table 1). These cognitive categories reflect the amount and direction of absolute change in DAS-II GCA score shown by patients during the study period: above-average, average, and below-average cognitive growth rates, plateauing cognitive functioning, or deteriorating cognitive functioning. The proportions of patients in the five cognitive categories were compared between treatment groups at 1 year (52 weeks; end of the phase 2/3 trial) and 2 years (combined data from the phase 2/3 trial and extension; 100-week visit for the early idursulfase-IT group and 104-week visit for the delayed idursulfase-IT group).

**Table 1:** Cognitive categories defined by baseline DAS-II GCA score, endpoint GCA score, and PRAS

Cognitive category	Description	Definition^a^
Above-average cognitive growth rate	Absolute and relative improvement, at a faster rate than same-age developmental norms	Endpoint GCA score − baseline GCA score > 10
Average cognitive growth rate	Absolute improvement, with relative improvement at the same rate as same-age developmental norms (stable GCA relative to norms)	Endpoint GCA score − baseline GCA score between −10 and 10
Below-average cognitive growth rate	Absolute improvement at a slower rate than same-age developmental norms, reflecting a relative decline (compared with normative development)	Endpoint GCA score − baseline GCA < −10 AND endpoint GCA score—PRAS > 10
Plateauing cognitive functioning	No absolute change, reflecting a relative decline (compared with normative development)	Endpoint GCA score − baseline GCA score < −10 AND endpoint GCA score—PRAS between −10 and 10
Deteriorating cognitive functioning	Absolute and relative decline	Endpoint GCA score − baseline GCA score) < −10 AND endpoint GCA score—PRAS < −10

^a^Seven patients younger than 3.5 years old at baseline transitioned from the DAS-II Early Years battery levels from the Early Years Lower Level (2.5–< 3.5 years; four subtests) to the Early Years Upper Level (3.5–< 7 years; six subtests) during the study; for these patients, developmental changes were evaluated based on a prorated GCA score at follow-up visits based on the four subtests in the Early Years Lower Level

*DAS-II* Differential Ability Scales, Second Edition; *GCA* General Conceptual Ability; *PRAS* Projected Retained Ability Score

DAS-II GCA scores over the 2 years were reported for patients in each of the five cognitive categories.

### Statistical analyses

The presented analyses are descriptive. To demonstrate the utility of PRAS in characterizing absolute change in the study samples over time, differences in the distribution of cognitive development categories between treatment groups in the overall population and in the subpopulation younger than 6 years of age were assessed using a post hoc, non-parametric, one-sided Mann–Whitney U-test. No multiplicity adjustments were made; therefore, these results are considered descriptive. Mean GCA scores over time for patients in each cognitive development category are presented graphically.

## Results

In total, 49 patients were randomly assigned to receive treatment in the phase 2/3 trial (idursulfase-IT, n = 34; no idursulfase-IT, n = 15), as described previously [20]. The two groups were well balanced in terms of baseline demographics and disease characteristics (Table 2). At the phase 2/3 trial baseline, nine patients were 6 years of age or older and 40 were younger than 6 years. Two of 49 patients discontinued the phase 2/3 trial (Additional file 2: Fig. S2) and did not enter the extension study (both in the idursulfase-IT group); there were no discontinuations in the first year of the extension study. Of 47 patients who entered the extension, 39 were assessed with the Early Years battery throughout the 2-year period, and two were assessed using the DAS-II School Age battery throughout. Six patients transitioned from the Early Years to the School Age battery during the 2-year follow-up: PRAS was calculated for two patients who were younger than 9 years old at the time of the School Age battery assessment; data from four patients who were 9 years of age or older at the time of the School Age battery assessment were excluded because PRAS could not be calculated (see Methods).

**Table 2:** Demographics and baseline characteristics

	Idursulfase-IT (n = 34)	No idursulfase-IT (n = 15)	Overall (N = 49)
*Age, years*			
Mean (SD)	5.0 (1.5)	5.3 (2.6)	5.1 (1.9)
Median (range)	4.6 (3.1, 8.7)	4.8 (3.1, 13.0)	4.6 (3.1, 13.0)
*Patients by age group, n (%)*			
Aged < 6 years	28 (82.4)	12 (80.0)	40 (81.6)
Aged ≥ 6 years	6 (17.6)	3 (20.0)	9 (18.4)
*Race, n (%)*			
White	23 (67.6)	12 (80.0)	35 (71.4)
Asian	4 (11.8)	0	4 (8.2)
Black or African American	1 (2.9)	0	1 (2.0)
Other	6 (17.6)	3 (20.0)	9 (18.4)
*Height, cm*			
Mean (SD)	111.7 (9.5)	110.9 (11.9)	111.5 (10.2) 108.8 (93.0, 140.0)
Median (range)	109.4 (95.7, 140.0)	107.9 (93.0, 137.7)	
*Weight, kg*			
Mean (SD)	24.5 (4.9)	25.3 (8.4)	24.8 (6.1)
Median (range)	23.8 (18.5, 39.8)	23.3 (17.0, 48.4)	23.6 (17.0, 48.4)
*Baseline DAS-II GCA score*			
Mean (SD)	68.4 (8.3)	67.3 (7.5)	68.0 (8.0)
Median (range)	67.5 (55, 85)	66.0 (56, 78)	67.0 (55, 85)
*Patients by baseline DAS-II GCA score category, n (%)*			
DAS-II GCA score ≤ 70	20 (58.8)	9 (60.0)	29 (59.2)
DAS-II GCA score > 70	14 (41.2)	6 (40.0)	20 (40.8)
*Patients by type of IDS variant, n (%)*			
Missense	17 (50.0)	7 (46.7)	24 (49.0)
Frameshift	5 (14.7)	3 (20.0)	8 (16.3)
Large deletion or complete deletion/large rearrangement	5 (14.7)	0	5 (10.2)
Intronic	2 (5.9)	2 (13.3)	4 (8.2)
Nonsense	3 (8.8)	1 (6.7)	4 (8.2)
Splice site	1 (2.9)	0	1 (2.0)
Unclassifiable	1 (2.9)	2 (13.3)	3 (6.1)

*DAS-II* Differential Ability Scales, Second Edition; *GCA* General Conceptual Ability; *IDS*, iduronate-2-sulfatase gene; *IT* intrathecal; *SD* standard deviation

Cognitive development categories (Table 1) were therefore assigned for 29/34 and 15/15 patients in the idursulfase-IT and no idursulfase-IT treatment groups, respectively, at 1 year, and for 27/32 and 12/15 patients in the early idursulfase-IT and delayed idursulfase-IT groups, respectively, at 2 years (Additional file 2: Fig. S2).

### Distribution of cognitive development categories

After 1 year, at the end of the phase 2/3 trial, there were numerically greater proportions of patients with above-average or average cognitive growth rate in DAS-II GCA scores in the idursulfase-IT group than in the no idursulfase-IT group (above-average: 6.9% [n = 2] vs. 0%; average: 65.5% [n = 19] vs. 53.3% [n = 8]; Table 3). The proportion of patients with deteriorating cognitive functioning was numerically lower in the idursulfase-IT group than in the no idursulfase-IT group (6.9% [n = 2] vs. 20.0% [n = 3]; Table 3).

**Table 3:** Cognitive categories^a^ at the end of year 1 and year 2

	Overall population		Subpopulation (aged < 6 years at baseline)	
	Idursulfase-IT in RCT (n = 34)	No idursulfase-IT in RCT (n = 15)	Idursulfase-IT in RCT (n = 28)	No idursulfase-IT in RCT (n = 12)
Cognitive category	Year 1 (n = 29)	Year 1 (n = 15)	Year 1 (n = 25)	Year 1 (n = 12)
Above-average cognitive growth rate	2/29 (6.9%)	0	2/25 (8.0%)	0
Average cognitive growth rate	19/29 (65.5%)	8/15 (53.3%)	17/25 (68.0%)	6/12 (50.0%)
Below-average cognitive growth rate	0	0	0	0
Plateauing cognitive functioning	6/29 (20.7%)	4/15 (26.7%)	5/25 (20.0%)	4/12 (33.3%)
Deteriorating cognitive functioning	2/29 (6.9%)	3/15 (20.0%)	1/25 (4.0%)	2/12 (16.7%)

Cognitive category	Year 2 (n = 27)	Year 2 (n = 12)	Year 2 (n = 25)	Year 2 (n = 11)
Above-average cognitive growth rate	1/27 (3.7%)	0	1/25 (4.0%)	0
Average cognitive growth rate	12/27 (44.4%)	5/12 (41.7%)	11/25 (44.0%)	4/11 (36.4%)
Below-average cognitive growth rate	1/27 (3.7%)	1/12 (8.25%)	1/25 (4.0%)	1/11 (9.1%)
Plateauing cognitive functioning	10/27 (37.0%)	5/12 (41.7%)	9/25 (36.0%)	5/11 (45.5%)
Deteriorating cognitive functioning	3/27 (11.1%)	1/12 (8.25%)	3/25 (12.0%)	1/11 (9.1%)

^a^Patients with a missing cognitive development category were omitted. There was no statistically significant difference between treatment groups for cognitive category distribution at 1 year (*p* = 0.0731) or 2 years (*p* = 0.4426) in the overall population. In the subpopulation aged < 6 years at baseline, there was a marginally significant difference between treatment groups for cognitive category distribution at 1 year (*p* = 0.0480) but no statistically significant difference at 2 years (*p* = 0.8579)

*IT* intrathecal; *RCT* randomized controlled trial

In the extension study, at 2 years, the proportions of patients across the cognitive categories were similar in both treatment groups; in the early idursulfase-IT group, 48.1% (n = 13) had above-average or average cognitive growth rates in DAS-II GCA scores compared with 41.7% of patients (n = 5) with average cognitive growth rates in DAS-II GCA scores in the delayed idursulfase-IT group (Table 3).

**Fig. 1:**
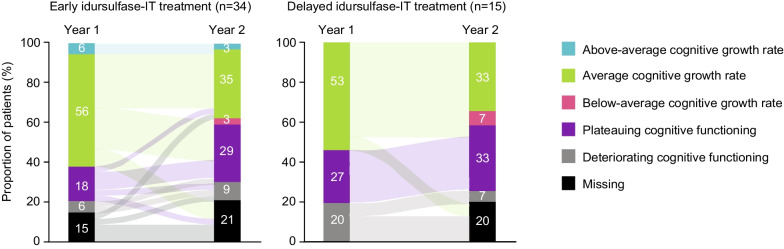
Distribution of, and patient movement between, cognitive categories in the overall population. Graph depicts the movement of individual patients from their cognitive category at 1 year to their cognitive category at 2 years; the width of each band corresponds to the number of patients who changed cognitive category with that movement. *IT* intrathecal

From 1 to 2 years, there was a decrease in the proportions of patients with above-average or average cognitive growth rates in DAS-II GCA scores in both treatment groups (Fig. 1). The majority of patients, however, remained in the same cognitive category at 1 and 2 years.

In the overall population, there was no statistically significant difference between treatment groups in the distributions of cognitive categories at either 1 year (*p* = 0.0731) or 2 years (*p* = 0.4426).

### Distributions of cognitive development categories (patients younger than 6 years of age)

In the subpopulation of patients younger than 6 years of age at phase 2/3 baseline, cognitive categories were defined for 25/28 and 12/12 patients in the idursulfase-IT and no idursulfase-IT groups, respectively, at 1 year, and for 25/28 and 11/12 patients in the early idursulfase-IT and delayed idursulfase-IT groups, respectively, at 2 years (Additional file 2: Fig. S2).

**Fig. 2:**
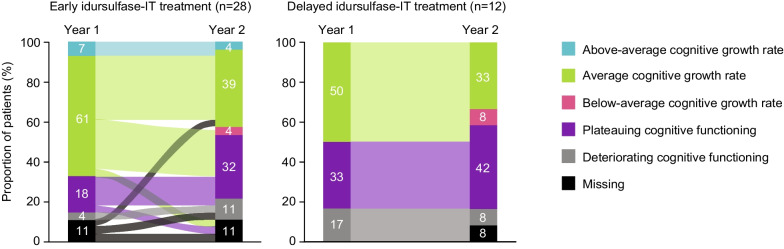
Distribution of, and patient movement between, cognitive categories in the subpopulation aged < 6 years. Graph depicts the movement of individual patients from their cognitive category at 1 year to their cognitive category at 2 years; the width of each band corresponds to the number of patients who changed cognitive category with that movement. *IT* intrathecal

Results for this subpopulation were similar to those for the overall population (Fig. 2). At 1 year, there were greater proportions of patients with above-average or average cognitive growth rate in DAS-II GCA scores in the idursulfase-IT group than in the no idursulfase-IT group (above-average: 8.0% [n = 2] vs. 0%; average: 68.0% [n = 17] vs. 50.0% [n = 6]; Table 3). The proportion of patients with deteriorating cognitive functioning at 1 year was 4.0% (n = 1) in the idursulfase-IT group and 16.7% (n = 2) in the no idursulfase-IT group (Table 3). The difference in distributions of cognitive categories with idursulfase-IT versus no idursulfase-IT at 1 year was statistically significant (*p* = 0.0480).

At 2 years, the proportions of patients in the different cognitive categories were more similar in the two treatment groups than at 1 year, with no statistically significant difference in category distributions between groups (*p* = 0.8579).

### DAS-II GCA scores within cognitive development categories

**Fig. 3:**
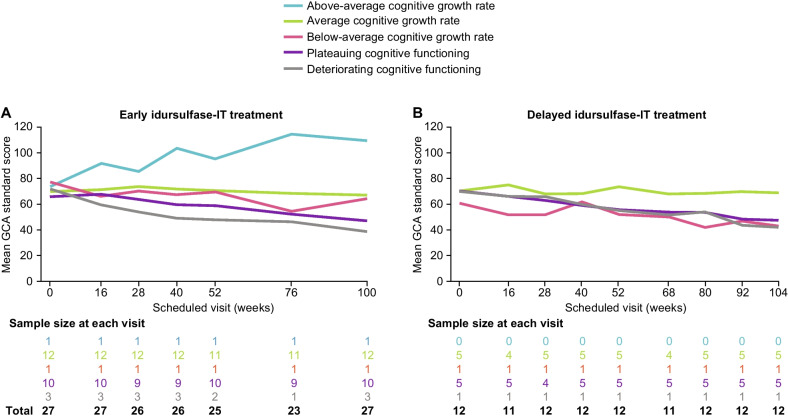
DAS-II GCA score^a^ by cognitive category in patients starting idursulfase-IT in the **A** RCT^b^ and **B** extension study. ^a ^Patients without data available for year 2 (week 100 or week 104) are not shown. ^b ^Patients who received treatment with idursulfase-IT in the RCT began the extension study during the extended treatment phase, which reflected a reduced frequency of assessments compared with the schedule in the RCT (neurodevelopmental assessments performed every 24 weeks). *DAS-II* Differential Ability Scales, Second Edition; *GCA* General Conceptual Ability; *IT* intrathecal; *RCT* randomized controlled trial

The one patient with above-average cognitive growth rates in DAS-II GCA scores (who received idursulfase-IT in the randomized trial) had an increasing trend in GCA score over the 2 years; there was a relatively flatter trend in GCA scores among patients with average cognitive growth rates in DAS-II GCA scores, and a decreasing trend in GCA scores in patients with below-average cognitive growth rates in DAS-II GCA scores, plateauing cognitive functioning, or deteriorating cognitive functioning (both treatment groups; Fig. 3A and B). Similar results were observed in the subpopulation younger than 6 years of age at baseline.

## Discussion

PRAS methodology has been developed to overcome the limitations of norm-based ability test scores in characterizing absolute changes over time in cognitive development [26]. This is of potential benefit in setting expectations, making treatment decisions, and determining treatment effects. In these post hoc analyses, PRAS methodology was applied to DAS-II GCA scores from the idursulfase-IT phase 2/3 trial and extension study. This allowed differentiation of patients who showed declines in norm-based DAS-II GCA scores into three separate categories (below-average cognitive growth rates in DAS-II GCA scores, cognitive development plateauing, and deterioration of cognitive functioning), in addition to patients who showed stable DAS-II GCA scores (average cognitive growth) and those who showed improvements in DAS-II GCA scores (above-average cognitive growth rates). This more specific differentiation of change in cognitive functioning over time during treatment suggests that PRAS methodology can be utilized to improve the understanding of changes in cognitive scores, particularly in patient populations who are at risk of deteriorating or plateauing cognitive performance. In those with declining standard cognitive scores, PRAS methodology may be a useful tool to help to interpret cognitive scores and to communicate results with parents and/or caregivers.

After 1 year of treatment, the idursulfase-IT group included a greater proportion of patients with above-average or average cognitive growth rates in DAS-II GCA scores and a lower proportion of patients with deteriorating cognitive functioning than the no idursulfase-IT group. Differences in the distribution of cognitive categories at 1 year did not reach statistical significance in the overall population but did reach statistical significance for the subpopulation aged younger than 6 years at phase 2/3 baseline in this post hoc analysis. Importantly, PRAS analyses allowed the differentiation of patients with declines in GCA scores after 1 year of treatment into the three categories of below-average, plateauing, and deteriorating cognitive functioning, revealing that approximately three times as many patients (proportionally) in the no idursulfase-IT group had deteriorating cognitive functioning after 1 year than patients in the idursulfase-IT group (Table 3; 20.0% vs. 6.9%); these values were even more pronounced in the baseline subsample of children younger than 6 years of age (16.7% vs. 4.0%). Although the overall number of patients is too low for statistical testing and for drawing firm conclusions from these data, the differences provide a conceptual example of the value of PRAS. Indeed, the significance of the difference in distributions of cognitive categories with idursulfase-IT versus no idursulfase-IT at 1 year was only borderline significant (*p* = 0.0480). It should be noted that the identification of participants in the deteriorating subgroups would not be possible using GCA scores alone.

At 2 years, both treatment groups were receiving idursulfase-IT in the extension study. Differences between treatment groups were less pronounced at 2 years than at 1 year, and did not reach statistical significance in either the overall population or the subpopulation younger than 6 years of age. However, many patients remained in the same cognitive category from 1 to 2 years.

The findings from PRAS analyses support and extend the results from the primary analyses of the phase 2/3 trial and extension study [20, 21]. In the phase 2/3 trial, the primary endpoint was not met in the overall population, but there were trends towards potential benefits of idursulfase-IT on DAS-II GCA scores, particularly in patients younger than 6 years of age at baseline. A post hoc subgroup analysis revealed a significant, clinically relevant treatment benefit on changes from baseline in DAS-II GCA score in those younger than 6 years of age at baseline with missense variants of *IDS* [20]. The smaller changes from baseline in DAS-II GCA scores observed in the early idursulfase-IT group compared with those in the delayed idursulfase-IT group, among patients younger than 6 years of age at baseline, were maintained for 36 months in the extension study [21]. There was also a more pronounced treatment benefit in those younger than 6 years of age with missense variants [21].

An analysis of the same trial and extension study data that categorized patients younger than 6 years old at baseline into three cognitive development categories (above-average, average, or below-average) based on their DAS-II GCA scores alone has also been reported previously [21]. In that analysis, the below-average developmental category included all patients with a decline in DAS-II GCA score of > 10 points. This category combined the PRAS categories of below-average cognitive growth rates in DAS-II GCA scores, plateauing cognitive functioning, and deteriorating cognitive functioning, without differentiating between them. Of note, in the early idursulfase-IT group from that analysis, greater proportions of patients had above-average or average cognitive development than in the delayed idursulfase-IT group at 1 year (76% vs. 50%) and 2 years (48% vs. 30%). Furthermore, a significant proportion of patients were included in the below-average category (52% and 70% in the early and delayed idursulfase-IT groups, respectively, at 2 years), and that might have been interpreted as a lack of treatment response. However, further analysis of those data using PRAS methodology revealed that only a small proportion of patients in the below-average category actually had deteriorating cognitive function at 2 years. Indeed, many patients in both the early and delayed idursulfase-IT groups achieved stabilization with treatment; this is a positive result in the context of this disease, although longer follow-up is required. This present analysis demonstrates the value of using PRAS methodology to understand the type of absolute change (Table 1) underlying a decline in norm-based cognitive ability test scores such as the DAS-II GCA.

Differences in the proportions of patients with missing data from 1 to 2 years in the two treatment groups may have affected the results. For example, the increase in the proportion of patients with missing data from 1 to 2 years in the delayed idursulfase-IT group mainly comprised patients who had been in the deteriorating cognitive category at 1 year, leading to a possible misinterpretation that the proportion of patients with deteriorating cognitive function had declined. However, approximately half of the patients in the early idursulfase-IT group and most of those in the delayed idursulfase-IT group remained in the same cognitive category at 1 year and 2 years. Moreover, patients typically moved to an adjacent category, with few patients having substantial changes in their cognitive status. The cognitive category with the greatest proportion of patients was that in which DAS-II GCA score cognitive growth rates were average (i.e., they were matching the trajectory of age-matched norms), and this remained the case at 1 year and 2 years.

These results should be considered in the context of current knowledge of the natural history of neuronopathic MPS II [10, 28–32]. Acknowledging the interpatient variability in cognitive development in neuronopathic MPS II, studies have described an early onset of developmental delay followed by a plateau of mental age/developmental skills at 3–8 years of age and a rapid decline thereafter [10, 28, 29, 31, 33]. Based on these findings, these children aged 3–13 years enrolled in the phase 2/3 trial may have been expected to experience deterioration in cognitive functioning over the 2 years. It is therefore of note that, in the present analysis using PRAS, the majority of patients remained in the same category at 1 year and 2 years post-baseline. The ability to document these rates of deterioration compared with stabilization or below-average cognitive growth rates over time in neuronopathic MPS II is an advantage of PRAS that is not possible using GCA or other norm-based scores alone.

Other limitations of the present analysis include those inherent to all post hoc analyses. The duration of follow-up, small sample sizes, and missing data for some patients, particularly at the 2-year timepoint, limit the conclusions that can be drawn regarding patterns and differences between groups and over time. While we considered a difference of > 10 points between endpoint and baseline GCA scores (relative change) or between endpoint GCA score and PRAS (absolute change) to be clinically and statistically meaningful based on our previous analysis, we acknowledge the limitations of this cut-off-based method, which should be taken into account in its application [26]. Specifically, deriving categories from continuous scores results in loss of some of the advantages of interval-level data in the statistical analysis and characterization of results. On the other hand, categories can be helpful for characterizing the functional/clinical meaning of scores and for communicating results to stakeholders. Because our primary intent was to illustrate the use of PRAS as a tool to characterize change in cognitive functioning over time, this was a descriptive analysis only with no adjustments for multiplicity testing in the statistical tests. Furthermore, a control group who did not receive idursulfase-IT was only included in the first year of study. We should acknowledge the apparent limited decline seen in the first year of the trial in these patients who were not receiving idursulfase-IT. This is consistent with the possibility that some patients had not yet reached a plateau in cognitive functioning at the time of enrolment into the trial. Also, as previously described, selective attrition may have influenced the 2-year data owing to disproportionate discontinuation of patients with deteriorating cognitive function at year 1 in the delayed idursulfase-IT group. In addition, floor or ceiling effects on DAS-II GCA scores could impact the value of the PRAS methodology by limiting the possible magnitude of differences between baseline and follow-up GCA scores [26]. Indeed, in the current analysis, some of the PRAS values were approaching floor scores for DAS-II GCA, limiting interpretation of the comparisons with these values. Furthermore, the assessment schedule for DAS-II GCA included testing every 12 weeks for both groups, which may have resulted in a mild elevation of score due to practice effects; however, because all patients had the same testing schedule, any effects were likely to be balanced between treatment groups.

It is also important to understand the meaning and value of PRAS in the context of other types of scores from cognitive ability tests. Although norm-based scores cannot be used in isolation to evaluate absolute change, other types of scores such as raw scores or item-response theory (IRT)-based scores (e.g., Rasch-scaled scores; referred to as ability scores for the DAS-II subtests) can be used to evaluate absolute change in individual or sample scores over time [22]. Rasch (IRT)-scaled ability scores are more advantageous than raw scores for this use because the level of difficulty is reflected in the item score value, whereas raw scores give each item the same value (e.g., 1 point).

Change in raw or IRT-based scores corresponds directly to participant performance on items within a subtest and is straightforward to calculate. However, unlike PRAS (which have meaning based on norm values), raw or IRT-based scores do not have inherent functional or clinical meaning. Thus, reliable and meaningful within-patient change values for raw or IRT-based scores would need to be established for the use of a subtest as a primary endpoint in a clinical trial (and would differ from one subtest to another) [34–37]. As a result, developing an endpoint for cognitive decline in MPS II using raw or IRT-based scores would require substantial evidence to support both (1) the use of a single subtest as a primary endpoint, and (2) the identification of values for meaningful within-patient change using a much larger sample size than can be achieved in ultra-rare diseases such as MPS II. PRAS, raw, or IRT-based score change analyses each have advantages and are not mutually exclusive or incompatible [34–37]. Hence, they could be used together when raw or IRT-based scores are available for a subtest to provide a more comprehensive evaluation of score change. However, because DAS-II GCA scores are not represented by raw or IRT-based scores, using raw or IRT-based scores to supplement PRAS change analyses of DAS-II GCA scores is not possible.

Future large-scale, long-term studies are required to fully assess the role of PRAS methodology in assessing specific profiles of cognitive function in patients with neurodegenerative conditions such as neuronopathic MPS II. When assessing cognitive function over the long term (> 24 months, i.e., a longer time period than in the present study), a combination of PRAS methodology and other established approaches is likely to be required for optimal interpretation of cognitive scores [26].

## Conclusion

The results from this analysis demonstrate that application of PRAS methodology to DAS-II GCA scores can be used to characterize change in cognitive functioning more specifically in patients with early-onset cognitive impairment reporting a decrease in norm-based scores. PRAS methodology allowed differentiation of patients who were considered to have below-average improvement in cognitive functioning (i.e., declines in GCA scores) into three separate categories reflecting absolute change in cognitive functioning (below-average cognitive development, plateauing cognitive functioning, and deteriorating cognitive functioning). Following 1 year of treatment with idursulfase-IT, a greater proportion of patients had above-average or average cognitive growth rate compared with those who had not received idursulfase-IT treatment, and a smaller proportion of patients who had received idursulfase-IT showed deteriorating cognitive functioning, particularly in patients younger than 6 years of age at phase 2/3 baseline. The differences between treatment groups were less pronounced at 2 years than at 1 year. Compared with a previously reported categorical analysis of the trial data, in which a high proportion of patients younger than 6 years of age at phase 2/3 baseline were categorized as having below-average cognitive development after treatment (reflected by a decline in DAS-II GCA scores), the present analysis demonstrated that only a small proportion of these patients actually had below-average cognitive growth rates in DAS-II GCA scores and, importantly, that the majority had plateauing cognitive functioning and remained in the same cognitive category. These results support further investigation into the role of PRAS methodology in assessing cognitive functioning over time in patients with neuronopathic MPS II.

### Supplementary Information

**Additional file 1**. Supplementary Material 1.

**Additional file 2**. Supplementary Material 2.

## Data Availability

The datasets, including redacted study protocol, redacted statistical analysis plan, and individual participants’ data supporting the results reported in this article, will be made available within 3 months from initial request to researchers who provide a methodologically sound proposal. The data will be provided after its de-identification in compliance with applicable privacy laws, data protection, and requirements for consent and anonymization. For more information, see https://clinicaltrials.takeda.com/takedas-commitment?commitment=5 and https://vivli.org/ourmember/takeda/.

## Abbreviations

DAS-II: Differential Ability Scales, Second Edition
GCA: General Conceptual Ability
I2S: Iduronate-2-sulfatase
*IDS*: Iduronate-2-sulfatase gene
IRT: Item-response theory
IT: Intrathecal
MPS: Mucopolysaccharidosis
PRAS: Projected Retained Ability Score
RCT: Randomized controlled trial
SD: Standard deviation
