# Supplementary material for: A post hoc analysis of Projected Retained Ability Scores (PRAS) for the longitudinal assessment of cognitive functioning in patients with neuronopathic mucopolysaccharidosis II receiving intrathecal idursulfase-IT
Source: Orphanet J Rare Dis. 2023 Nov 2;18:343. doi: 10.1186/s13023-023-02957-2 (PMC10621086; doi:10.1186/s13023-023-02957-2)
Supplement: Supplementary file 1 — Additional file 1. Supplementary Material 1. [file 13023_2023_2957_MOESM1_ESM.pdf]

Supplementary Figure 1. Calculation method for the DAS-II GCA and PRAS GCA scores from raw scores at baseline and at follow-up.

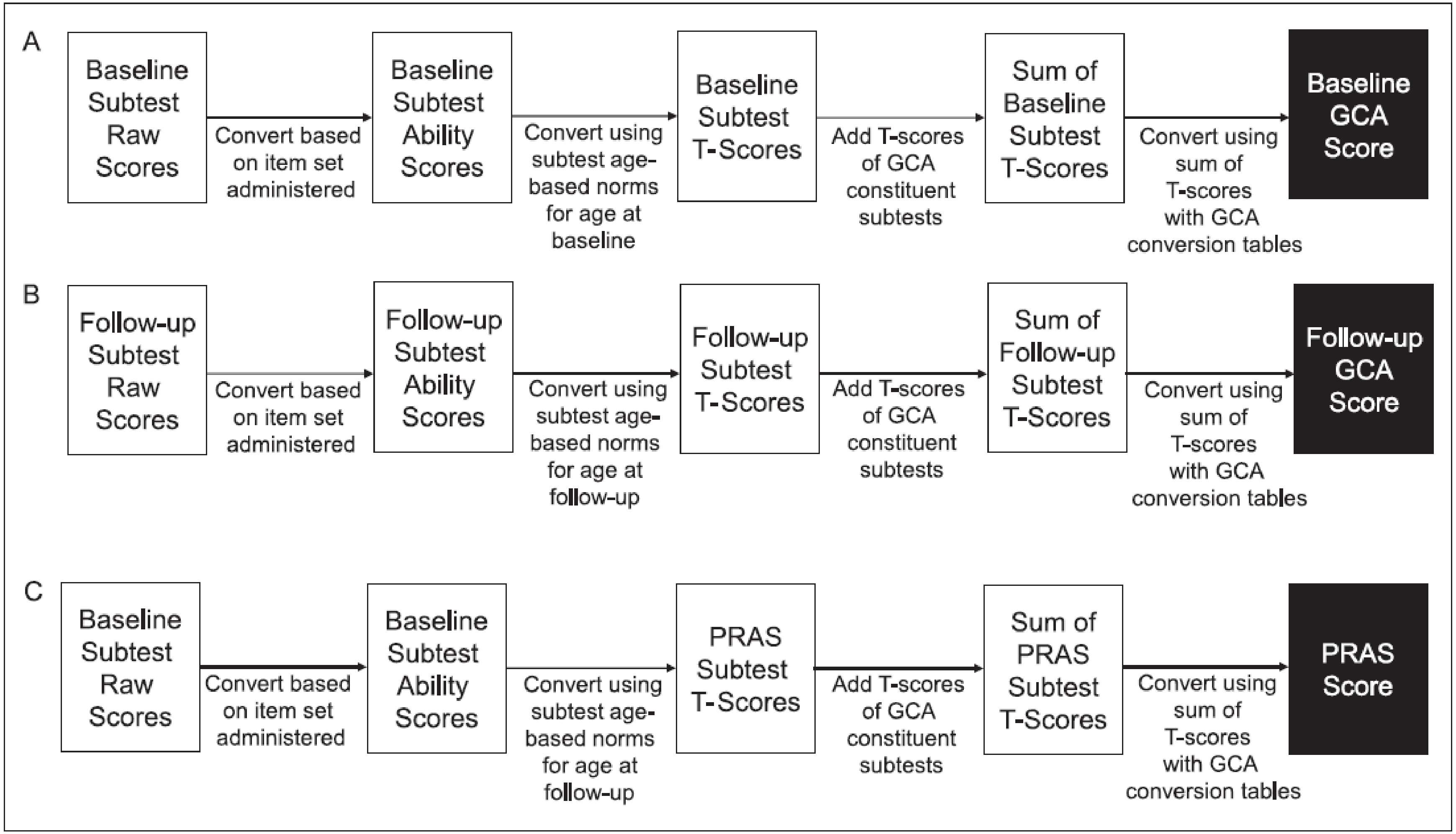

DAS-II, Differential Ability Scales, Second Edition; GCA, General Conceptual Ability; PRAS, Projected Retained Ability Score.
