# Supplementary material for: A post hoc analysis of Projected Retained Ability Scores (PRAS) for the longitudinal assessment of cognitive functioning in patients with neuronopathic mucopolysaccharidosis II receiving intrathecal idursulfase-IT
Source: Orphanet J Rare Dis. 2023 Nov 2;18:343. doi: 10.1186/s13023-023-02957-2 (PMC10621086; doi:10.1186/s13023-023-02957-2)
Supplement: Supplementary file 2 — Additional file 2. Supplementary Material 2. [file 13023_2023_2957_MOESM2_ESM.pdf]

Supplementary Figure 2. Patient disposition and DAS-II GCA data availability for the ITT and subpopulation of patients younger than 6 years of age at baseline.

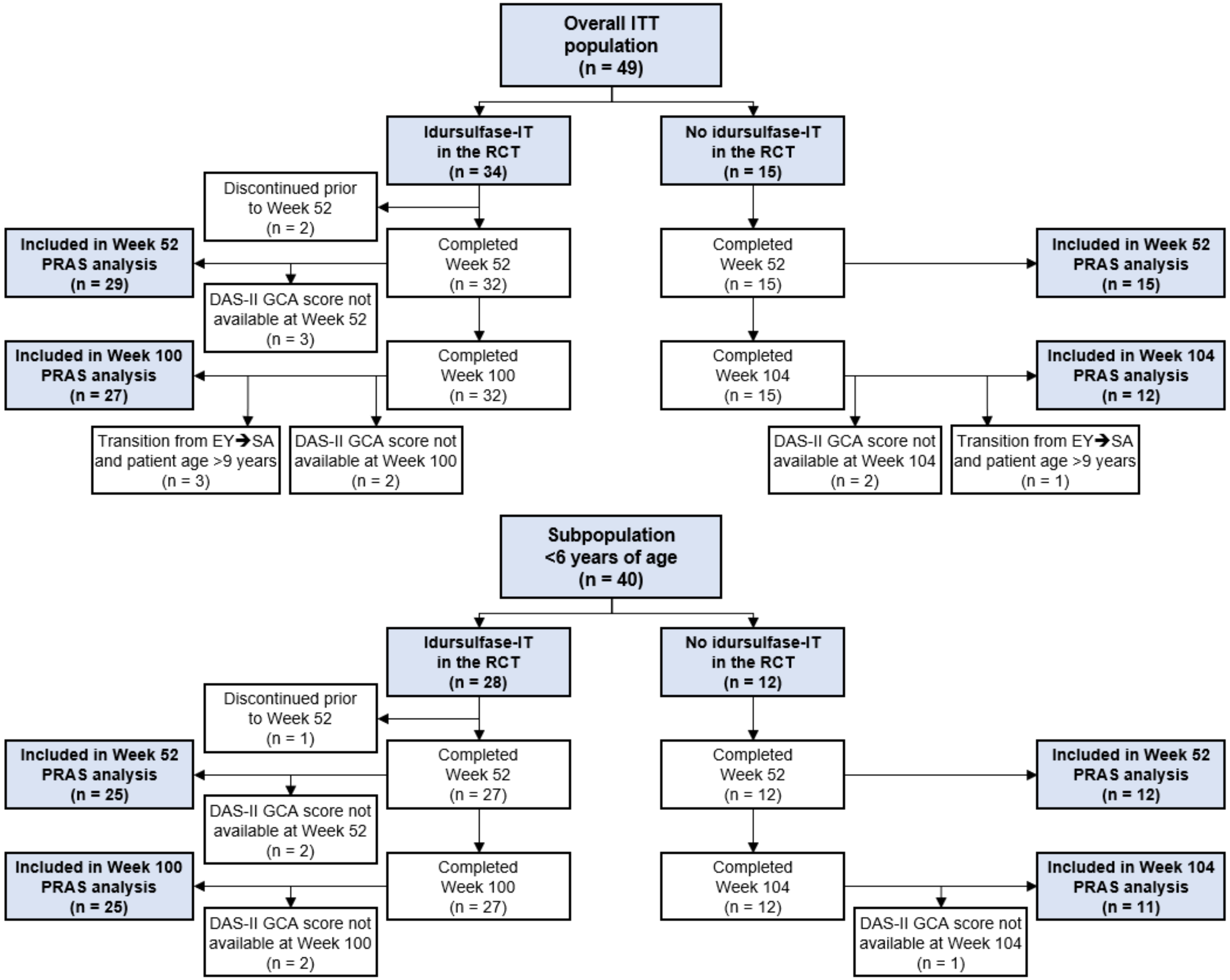

DAS-II, Differential Ability Scales, Second Edition; EY, early years; GCA, General Conceptual Ability; IT, intrathecal; ITT; intention-to-treat; RCT, randomized clinical trial; SA, school age.
